# Synthesis, optical and electrochemical properties of 4,4′-bibenzo[*c*]thiophene derivatives†

**DOI:** 10.1039/d1ra01189h

**Published:** 2021-05-25

**Authors:** Kotaro Obayashi, Keiichi Imato, Satoshi Aoyama, Toshiaki Enoki, Seiji Akiyama, Mio Ishida, Seiji Suga, Koichi Mitsudo, Yousuke Ooyama

**Affiliations:** Department of Applied Chemistry, Graduate School of Engineering, Hiroshima University 1-4-1 Kagamiyama Higashi-Hiroshima 739-8527 Japan yooyama@hiroshima-u.ac.jp +81 82-424-5494; Science & Innovation Center, Mitsubishi Chemical Corporation 1000, Kamoshida-cho, Aoba-ku, Yokohama-shi Kanagawa 227-8502 Japan; Division of Applied Chemistry, Graduate School of Natural Science and Technology, Okayama University 3-1-1 Tsushima-naka, Kita-ku Okayama 700-8530 Japan

## Abstract

We designed and synthesized unsubstituted 4,4′-bibenzo[*c*]thiophene 4,4′-BBT and its silyl-substituted derivatives 1,1′-Si-4,4′-BBT and 1,1′,3,3′-Si-4,4′-BBT with one or two *tert*-butyldimethylsilyl groups on each thiophene ring, as new π-building blocks in emitters, photosensitizers and semiconductors for organic optoelectronic devices. The characterization of 4,4′-BBT, 1,1′-Si-4,4′-BBT and 1,1′,3,3′-Si-4,4′-BBT was successfully determined by FTIR, ^1^H and ^13^C NMR measurements, high-resolution mass spectrometry (HRMS) analysis, photoabsorption and fluorescence spectroscopy, cyclic voltammetry (CV) and density functional theory (DFT) calculations. Moreover, a single-crystal X-ray structural analysis was successfully made for 1,1′-Si-4,4′-BBT and 1,1′,3,3′-Si-4,4′-BBT. The photoabsorption and fluorescence maxima (*λ*^abs^_max_ and *λ*^fl^_max_) of the three 4,4′-bibenzo[*c*]thiophene derivatives in toluene exhibit bathochromic shifts in the order of 4,4′-BBT (359 nm and 410 nm) < 1,1′-Si-4,4′-BBT (366 nm and 420 nm) < 1,1′,3,3′-Si-4,4′-BBT (371 nm and 451 nm). The HOMO and LUMO energy levels rise in the order of 4,4′-BBT (−5.55 eV and −2.39 eV) < 1,1′-Si-4,4′-BBT (−5.45 eV and −2.34 eV) < 1,1′,3,3′-Si-4,4′-BBT (−5.34 eV and −2.30 eV), but the rise of the HOMO energy level is larger than that of the LUMO energy level, resulting in the bathochromic shift of the photoabsorption band from 4,4′-BBT to 1,1′,3,3′-Si-4,4′-BBT. The fluorescence quantum yields (*Φ*_fl_) of 4,4′-BBT, 1,1′-Si-4,4′-BBT and 1,1′,3,3′-Si-4,4′-BBT in toluene are 0.41, 0.41 and 0.36, respectively. It is worth mentioning that in the solid state 1,1′-Si-4,4′-BBT and 1,1′,3,3′-Si-4,4′-BBT show relatively high *Φ*_fl-solid_ values of 0.22 and 0.25, respectively, whereas 4,4′-BBT exhibits poor solid-state fluorescence properties (*Φ*_fl-solid_ < 0.02). This work provides an efficient synthetic method for the 4,4′-bibenzo[*c*]thiophene derivatives and their photophysical properties in the solution and solid state, electrochemical properties and X-ray crystal structures.

## Abstract 2

4,4′-Bibenzo[*c*]thiophene 4,4′-BBT and its silyl-substituted derivatives 1,1′-Si-4,4′-BBT and 1,1′,3,3′-Si-4,4′-BBT have been designed and developed as new π-building blocks.

## Graphical abstract

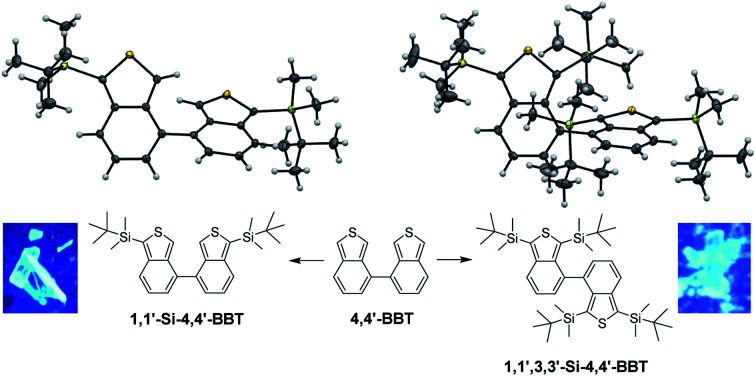

## Introduction

**Fig. 1:**
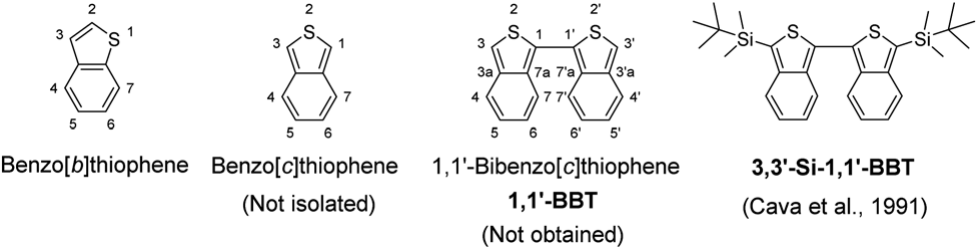
Chemical structures of benzo[*b*]thiophene, benzo[*c*]thiophene, 1,1′-bibenzo[*c*]thiophene 1,1′-BBT and 3,3′-silyl-disubstituted-1,1′-bibenzo[*c*]thiophene 3,3′-Si-1,1′-BBT.

**Fig. 2:**
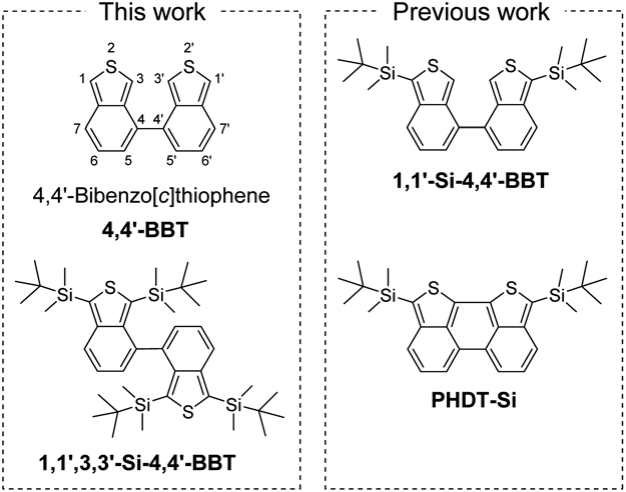
Chemical structures of 4,4′-bibenzo[*c*]thiophene 4,4′-BBT (BBT-1), 1,1′-silyl-disubstituted-4,4′-bibenzo[*c*]thiophene 1,1′-Si-4,4′-BBT (BBT-2), 1,1′,3,3′-silyl-tetrasubstituted-4,4′-bibenzo[*c*]thiophene 1,1′,3,3′-Si-4,4′-BBT (BBT-3) and fused-bibenzo[*c*]thiophene PHDT-Si.

Benzo[*b*]thiophene as a π-building block has created considerable scientific interest in synthetic organic chemistry, photochemistry, electrochemistry and theoretical chemistry as well as materials chemistry, because it is an especially crucial player for functional materials due to the air-stability and commercial availability (Fig. 1).^1^ Actually, benzo[*b*]thiophene derivatives are key constituents of emitters, semiconductors and photosensitizers for organic optoelectronic devices, such as organic light-emitting diodes (OLEDs),^2^ organic field-effect transistors (OFETs),^3^ organic photovoltaics (OPVs)^4^ and dye-sensitized solar cells (DSSCs).^5^ Furthermore, much effort has been made towards the construction and characterization of fused benzo[*b*]thiophene systems such as thienoacenes (*e.g.*, [1]benzothieno[3,2-*b*][1]benzothiophene (BTBT), dinaphtho[2,3-*b*:2′,3′-*f*]thieno[3,2-*b*]thiophene (DNTT) and dianthra[2,3-*b*:2′,3′-*f*]thieno[3,2-*b*]thiophene (DATT))^6*a*^ and thiophene-fused naphtho[2,3-*b*:6,7-*b*]dithiophene diimide (NDTI)^6*b*^ in the past two decades. Indeed, these benzo[*b*]thiophene derivatives have been used as organic semiconductors with high carrier mobility and stability under ambient conditions. Similarly, benzo[*c*]thiophene is also an interesting π-building block (Fig. 1). For example, Wudl and Heeger have prepared poly(1,3-benzo[*c*]thiophene) (*i.e.*, poly(isothianaphthene) (PITN)) and demonstrated that PITN can form the aromatic and quinoidal states by the bond alternation.^7^ In the aromatic state, the benzo[*c*]thiophene unit contains a thiophene ring in the structure. On the other hand, in the quinoidal state, the unit contains the more stable benzene ring in the structure. Thus, PITN has a low band gap (*E*_g_ = 1.0–1.2 eV), which is about 1.0 eV lower than that of polythiophene (PT), because the polymer backbone of PITN intrinsically stabilizes its quinoidal state, that is, the contribution of the quinoidal state decreases the *E*_g_. However, unsubstituted benzo[*c*]thiophene has never been isolated so far due to the instability in air, in contrast to unsubstituted benzo[*b*]thiophene. Nevertheless, some benzo[*c*]thiophene derivatives with substituents on the thiophene ring and/or the benzene ring such as 1,3-diarylbenzo[*c*]thiophenes,^8–12^ 5,6-disubstituted benzo[*c*]thiophenes^13^ and 1,3,4,7- or 1,3,5,6-tetrasubstituted benzo[*c*]thiophenes^14–16^ have been synthesized and their optical and electrochemical properties were investigated, although much less substituted benzo[*c*]thiophene derivatives have been reported than substituted benzo[*b*]thiophene derivatives. In particular, few 3,3′-disubstituted-1,1′-bibenzo[*c*]thiophenes as the 1,1′-dimer of bibenzo[*c*]thiophene have been developed by Cava,^17^ Mohanakrishnan^18^ and Ono *et al.*,^19^ and they revealed their synthetic methods and, optical and electrochemical properties, although there are no reports on the synthesis and physical properties of unsubstituted 1,1′-bibenzo[*c*]thiophene 1,1′-BBT (Fig. 1). On the other hand, in our previous work,^20^ we have designed and developed 1,1′-bis(*tert*-butyldimethylsilyl)-4,4′-bibenzo[*c*]thiophene (1,1′-Si-4,4′-BBT) as the 4,4′-dimer of bibenzo[*c*]thiophene and the fused-bibenzo[*c*]thiophene (PHDT-Si), which is the first report on the synthesis, characterization and optical and electrochemical properties of 4,4′-bibenzo[*c*]thiophene and fused-bibenzo[*c*]thiophene derivatives (Fig. 2). PHDT-Si exhibits intense vibronic-structured photoabsorption (*λ*^abs^_max_ = 598 nm, molar extinction coefficient (*ε*_max_) = 80 900 M^−1^ cm^−1^ in toluene) and fluorescence (*λ*^fl^_max_ = 613 nm, fluorescence quantum yield (*Φ*_fl_) = 0.74 in toluene) bands in a significantly longer wavelength region and a smaller Stokes shift (409 cm^−1^), compared to those of 1,1′-Si-4,4′-BBT (*λ*^abs^_max_ = 366 nm, *λ*^fl^_max_ = 420 nm, *ε*_max_ = 14 400 M^−1^ cm^−1^, *Φ*_fl_ = 0.41, Stokes shift = 3513 cm^−1^ in toluene). It is worth noting here that 1,1′-Si-4,4′-BBT exhibits the *λ*^abs^_max_ in a shorter wavelength region by 40 nm in comparison with that of the isomer 3,3′-bis(*tert*-butyldimethylsilyl)-1,1′-bibenzo[*c*]thiophene 3,3′-Si-1,1′-BBT (*λ*^abs^_max_ = 406 nm, *ε*_max_ = *ca.* 11 500 M^−1^ cm^−1^ in CH_2_Cl_2_) reported by Cava *et al.*^17*a*^ However, in order to use extensively and commonly benzo[*c*]thiophene derivatives as π-building blocks in the emitters, photosensitizers and semiconductors for organic optoelectronic devices, it is necessary to develop efficient and facile synthetic methods for benzo[*c*]thiophene derivatives.

Therefore, the aim of this work is to provide the synthetic strategy for 4,4′-bibenzo[*c*]thiophene derivatives and to reveal their optical and electrochemical properties. With this aim, we designed and synthesized unsubstituted 4,4′-bibenzo[*c*]thiophene (4,4′-BBT: abbr. as BBT-1) and its silyl-substituted derivatives 1,1′,3,3′-Si-4,4′-BBT (abbr. as BBT-3) with two sterically hindered *tert*-butyldimethylsilyl groups on each thiophene ring as well as 1,1′-Si-4,4′-BBT (abbr. as BBT-2) (Fig. 2). The characterization of BBT-1, BBT-2 and BBT-3 was successfully determined by FTIR, ^1^H and ^13^C NMR measurements, high-resolution mass spectrometry (HRMS) analysis, photoabsorption and fluorescence spectroscopy, cyclic voltammetry (CV) and density functional theory (DFT) calculations. This work is the first to achieve the synthesis, photophysical and electrochemical characteristics of unsubstituted 4,4′-bibenzo[*c*]thiophene BBT-1. Moreover, we achieved the single-crystal X-ray structural analysis of BBT-2 and BBT-3. Herein we report an efficient synthetic method for the 4,4′-bibenzo[*c*]thiophene derivatives and their photophysical properties in the solution and the solid state, electrochemical properties and X-ray crystal structures.

## Results and discussion

### Synthesis

**Scheme 1:**
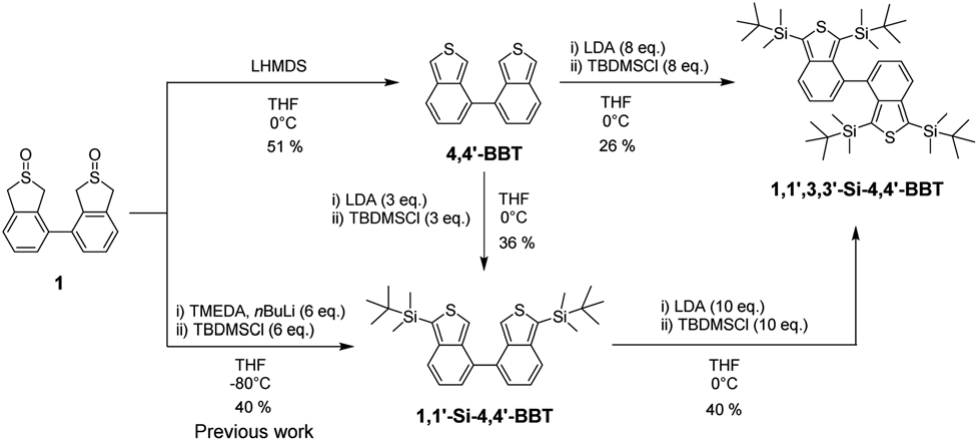
Synthetic route to 4,4′-bibenzo[*c*]thiophene derivatives 4,4′-BBT (BBT-1), 1,1′-Si-4,4′-BBT (BBT-2) and 1,1′,3,3′-Si-4,4′-BBT (BBT-3).

4,4′-Bibenzo[*c*]thiophene (BBT-1) and its silyl-substituted derivatives (BBT-2 and BBT-3) were synthesized according to a stepwise synthetic protocol (Scheme 1). Indeed, unsubstituted 4,4′-bibenzo[*c*]thiophene (BBT-1) was successfully prepared by treatment of 1,1′,3,3′-tetrahydro-[4,4′-bibenzo[*c*]thiophene] 2,2′-dioxide 1 (see ref. 20 for the synthesis) with lithium hexamethyldisilazide (LHMDS). In our previous work, we demonstrated that BBT-2 with a *tert*-butyldimethylsilyl group on each thiophene ring was obtained by the reaction of 1 with tetramethylethylenediamine (TMEDA) and then *n*BuLi, followed by treatment with *tert*-butyldimethylsilyl chloride (TBDMSCl).^20^ In this work, we found that BBT-2 was also prepared by the reaction of BBT-1 with lithium diisopropylamide (LDA), followed by treatment with TBDMSCl. It is worth noting here that the above reactions from 1 or BBT-1 did not yield 3,3′-Si-4,4′-BBT with two *tert*-butyldimethylsilyl groups at the 3,3′-positions. This result indicates that the lithiation of BBT-1 preferentially occurs at the 1,1′-positions rather than the 3,3′-positions. On the other hand, BBT-3 was not obtained directly from 1 even under the condition using TMEDA, *n*BuLi (10 eq.) and then TBDMSCl (10 eq.). Finally, we prepared BBT-3 with two *tert*-butyldimethylsilyl groups on each thiophene ring by the reaction of BBT-1 or BBT-2 with LDA, followed by treatment with TBDMSCl. The characterization of BBT-1, BBT-2 and BBT-3 was successfully determined by FTIR, ^1^H and ^13^C NMR measurements and HRMS analysis. Therefore, this result proposes the stepwise synthetic method for the introduction of substituents into the thiophene rings of the 4,4′-bibenzo[*c*]thiophene skeleton.

### X-ray crystal structures

**Fig. 3:**
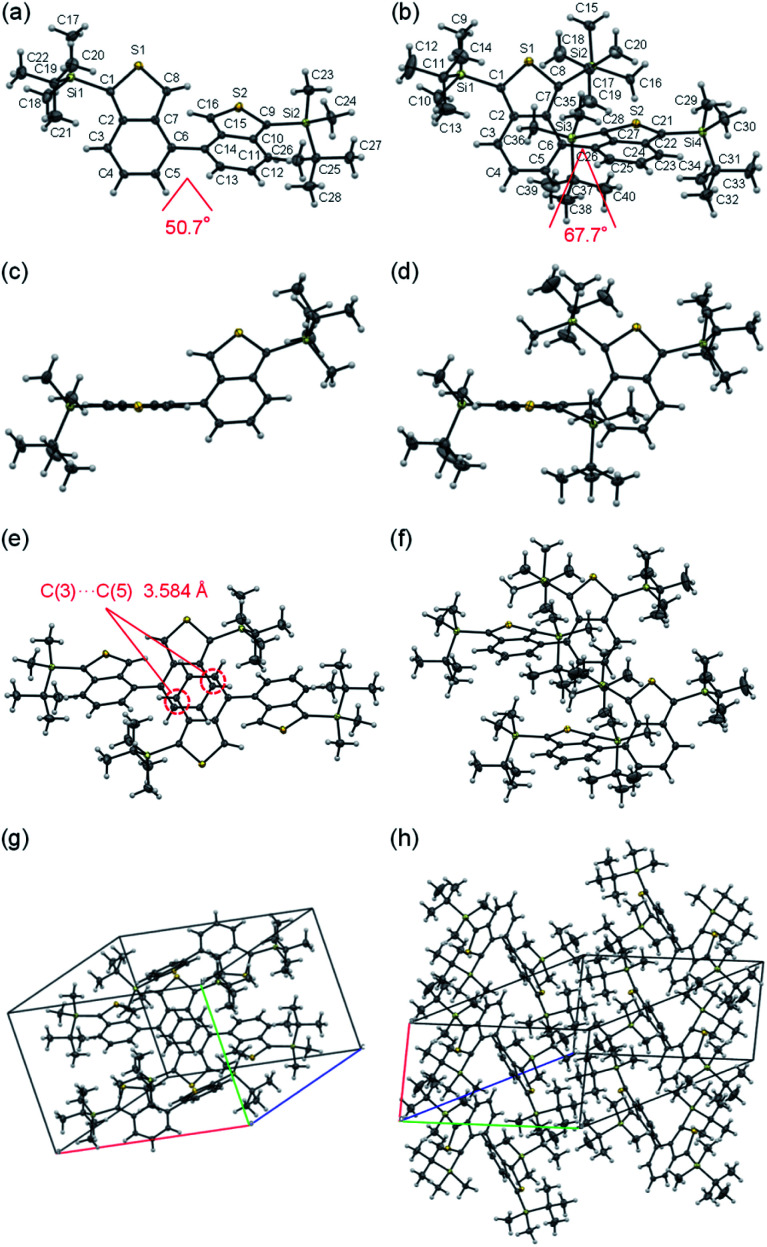
Crystal structures of 1,1′-Si-4,4′-BBT (BBT-2) and 1,1′,3,3′-Si-4,4′-BBT (BBT-3): (a) top view, (c) side view, (e) a top view of the pair of molecules and (g) molecular packing structure of BBT-2, and (b) top view, (d) side view, (f) a top view of the pair of molecules and (h) molecular packing structure of BBT-3.

A single-crystal X-ray structural analysis was successfully made for BBT-2 and BBT-3 (Fig. 3), while unfortunately, we could not obtain single crystals of BBT-1 with sufficient size to make the X-ray structural analysis possible. The dihedral angles between the two benzo[*c*]thiophene units in BBT-2 and BBT-3 are 50.7° and 67.7° (Fig. 3a–d), respectively, which show that the two units in BBT-3 twist considerably due to the steric hindrance of the *tert*-butyldimethylsilyl groups at the 3,3′-positions, compared to those in BBT-2. The crystal structure of BBT-2 is made up of dimer units composed of pairs of molecules (Fig. 3e and g). There are two short interatomic contacts of less than 3.60 Å between a pair of molecules, that is, the interatomic distance between C(3) in a benzo[*c*]thiophene unit and C(5) in the other unit is *ca.* 3.58 Å. In the crystal structure of BBT-3, on the other hand, there are no short π–π contacts of less than 3.60 Å between the neighboring molecules (Fig. 3f and h), which indicates the absence of the π–π interactions between the molecules.

### Photophysical properties in the solution and the solid state

**Fig. 4:**
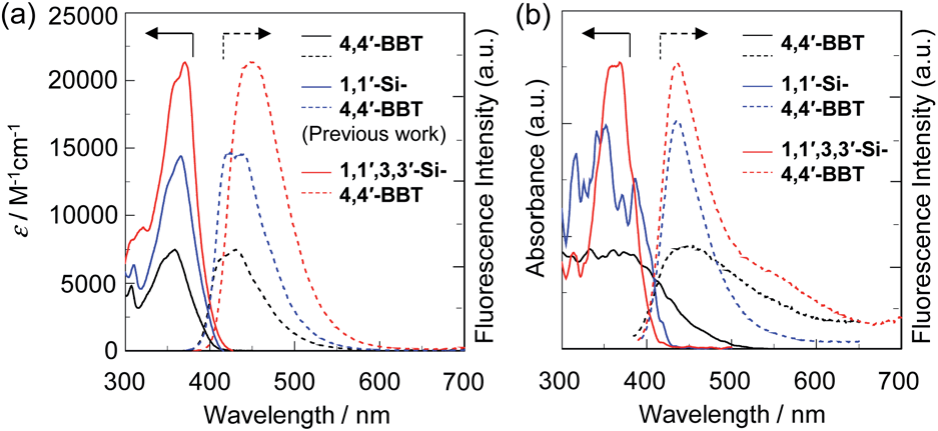
(a) Photoabsorption (solid line) and fluorescence (dotted line) spectra of 4,4′-BBT (BBT-1), 1,1′-Si-4,4′-BBT (BBT-2) and 1,1′,3,3′-Si-4,4′-BBT (BBT-3) in toluene. (b) Solid-state UV-vis diffuse reflection–absorption (solid line) and fluorescence (dotted line) spectra (*λ*^ex^ = 360 nm) of the as-recrystallized BBT-1, BBT-2 and BBT-3.

The photoabsorption and fluorescence spectra of BBT-1, BBT-2 and BBT-3 in toluene are shown in Fig. 4a, and their photophysical data are summarized in Table 1. The photoabsorption spectra demonstrated that BBT-2 and BBT-3 exhibit an intense photoabsorption band (*λ*^abs^_max_ = 366 nm and 371 nm, respectively) with a relatively high *ε*_max_ value (14 400 M^−1^ cm^−1^ and 21 300 M^−1^ cm^−1^, respectively) in a longer wavelength region by 7 nm and 12 nm, respectively, in comparison with that of BBT-1 (*λ*^abs^_max_ = 359 nm, *ε*_max_ = 7500 M^−1^ cm^−1^), due to the electron-donating *tert*-butyldimethylsilyl group. The corresponding fluorescent bands of the three fluorophores appear in longer wavelength regions in the order of BBT-1 (*λ*^fl^_max_ = 410 nm) < BBT-2 (*λ*^fl^_max_ = 420 nm) < BBT-3 (*λ*^fl^_max_ = 451 nm), and thus, the Stokes shift (SS) values increase in the order of BBT-1 (3465 cm^−1^) ≈ BBT-2 (3513 cm^−1^) < BBT-3 (4781 cm^−1^). The *Φ*_fl_ values of BBT-1, BBT-2 and BBT-3 are 0.41, 0.41 and 0.36, respectively, indicating moderate fluorescence properties. The relatively low *Φ*_fl_ and large SS values of BBT-3 indicate significant changes in the molecular and electronic structures between the ground and excited states by the rotation or twisting of the two benzo[*c*]thiophene units, due to the steric hindrance of the *tert*-butyldimethylsilyl groups at the 3,3′-positions. The time-resolved fluorescence spectroscopy of the three fluorophores demonstrated that the fluorescence lifetimes (*τ*_fl_) are 3.46 ns for BBT-1, 3.19 ns for BBT-2 and 3.59 ns for BBT-3, and thus, there are little difference in the *τ*_fl_ values between the three fluorophores. The radiative rate constant (*k*_r_ = 1.00 × 10^8^ s^−1^) for BBT-3 is slightly smaller than those (1.18 × 10^8^ s^−1^ and 1.29 × 10^8^ s^−1^, respectively) for BBT-1 and BBT-2. In contrast, the nonradiative rate constants (*k*_nr_ = 1.70–1.84 × 10^8^ s^−1^) of the three fluorophores resemble each other. Consequently, the ratio of nonradiative constant to radiative constant (*k*_nr_/*k*_r_ = 1.78) for BBT-3 is larger than those (1.44 and 1.43, respectively) for BBT-1 and BBT-2, suggesting that the lower *Φ*_fl_ value of BBT-3 is mainly due to the smaller *k*_r_ value compared to those of BBT-1 and BBT-2.

**Table 1:** Photophysical and electrochemical data and HOMO and LUMO energy levels of 4,4′-bibenzo[*c*]thiophene derivatives in the solution

Dye	*λ* ^abs^_max_ a/nm (*ε*_max_/M^−1^ cm^−1^)	*λ* ^fl^_max_ b/nm (*Φ*_fl_)	*τ*_fl_ c/ns	*k*_r_ d/s^−1^	*k*_nr_ e/s^−1^	*k*_nr_ */k*_r_	*E* ^ox^_onset_ f/V	*E* ^opt^_g_ g/eV	HOMO^h^/eV	LUMO^h^/eV
4,4′-BBT	359 (7500)	410 (0.41)	3.46	1.18 × 10^8^	1.70 × 10^8^	1.44	0.75	3.16	−5.55	−2.39
1,1′-Si-4,4′-BBT	366 (14 400)^i^	420 (0.41)^i^	3.19^i^	1.29 × 10^8^^i^	1.84 × 10^8^^i^	1.43^i^	0.65^i^	3.11^i^	−5.45^i^	−2.34^i^
1,1′,3,3′-Si-4,4′-BBT	371 (21 300)	451 (0.36)	3.59	1.00 × 10^8^	1.78 × 10^8^	1.78	0.54	3.04	−5.34	−2.30

a In toluene.

b In toluene. Fluorescence quantum yields (*Φ*_fl_) were determined by using a calibrated integrating sphere system (*λ*^ex^ = 359 nm, 366 nm and 371 nm for 4,4′-BBT (BBT-1), 1,1′-Si-4,4′-BBT (BBT-2) and 1,1′,3,3′-Si-4,4′-BBT (BBT-3), respectively).

c Fluorescence lifetime.

d Radiative rate constant (*k*_r_ = *Φ*_fl_/*τ*_fl_).

e Nonradiative rate constant (*k*_nr_ = (1 − *Φ*_fl_)/*τ*_fl_).

f Onset (*E*^ox^_onset_) *versus* Fc/Fc^+^ of the oxidation potential.

g Optical energy gaps (*E*^opt^_g_) were determined from the intersection (393 nm, 399 nm and 408 nm for BBT-1, BBT-2 and BBT-3, respectively) of photoabsorption and fluorescence spectra in toluene.

h *Versus* vacuum level.

i Previous work (ref. 20).

In order to investigate the solid-state photophysical properties of BBT-1, BBT-2 and BBT-3, we have measured the solid-state UV-Vis diffuse reflection–photoabsorption and fluorescence spectra for the solids (Fig. 4b), and their photophysical data are summarized in Table 2. Both BBT-2 and BBT-3 in the solid state show a photoabsorption band at around 360 nm with an onset at 420–425 nm, which is similar to the corresponding photoabsorption band of the two fluorophores in toluene (Fig. 4a). On the other hand, the photoabsorption band of BBT-1 in the solid state is broadened in a longer wavelength region with an onset of *ca.* 500 nm, in comparison with that in toluene. The corresponding solid-state fluorescence spectra revealed that BBT-1 and BBT-2 show a fluorescence band (*λ*^fl-solid^_max_ = 455 nm and 435 nm, respectively) in a longer wavelength region by 45 nm and 15 nm, respectively, compared to those in toluene. It is worth mentioning here that the fluorescence band (*λ*^fl-solid^_max_ = 435 nm) of BBT-3 in the solid state appeared in a shorter wavelength region than that (*λ*^fl^_max_ = 451 nm) in toluene (Fig. 4a). The *Φ*_fl-solid_ values of BBT-1, BBT-2 and BBT-3 in the solid state are <0.02, 0.22 and 0.25, respectively, which are lower than those in toluene (Table 1). In particular, the fluorescence properties of BBT-1 were strongly quenched in the solid state. The bathochromic shifts of *λ*^abs^_max_ and *λ*^fl^_max_ and the lowering of *Φ*_fl_ value by changing from the solution to the solid state are quite common and explained in terms of the formation of intermolecular π–π interactions between the fluorophores in the solid state and consequent delocalization of excitons or excimers.^21^ Thus, for BBT-1, the bathochromic shift of *λ*^fl^_max_ and the significant lowering of *Φ*_fl_ value by changing from the solution to the solid state would be attributed to the formation of intermolecular π–π interactions between the fluorophores in the solid state. On the other hand, the relatively high *Φ*_fl-solid_ value of BBT-3 in the solid state is based on the fact that the short π–π contacts of less than 3.60 Å between the neighboring molecules were not observed in the crystal structure of BBT-3 (Fig. 3f), which indicates the absence of the intermolecular π–π interactions between the fluorophores. Indeed, the four *tert*-butyldimethylsilyl groups at the 1,1′,3,3′-positions can effectively prevent the fluorophores from forming intermolecular π–π interactions in the solid state. In addition, for BBT-3, the hypsochromic shift of *λ*^fl^_max_ by changing from the solution to the solid state may be attributed to inhibition of structural relaxation in the excited state by restriction of the rotation or twisting of the two benzo[*c*]thiophenes in the solid state, due to the steric hindrance of the *tert*-butyldimethylsilyl groups at the 3,3′-positions between the neighboring fluorophores. The time-resolved fluorescence spectroscopy of the three 4,4′-bibenzo[*c*]thiophene derivatives in the solid state demonstrated that the *τ*_fl-solid_ values are 1.61 ns for BBT-2 and 2.33 ns for BBT-3, which are shorter than those (3.19 ns and 3.59 ns, respectively) in toluene. However, the precise evaluation of the *τ*_fl-solid_ value (<1 ns) of BBT-1 was difficult due to its feeble solid-state fluorescence properties. The *k*_r-solid_ values for BBT-2 and BBT-3 in the solid state are 1.38 × 10^8^ s^−1^ and 9.00 × 10^7^ s^−1^, respectively, which are almost equivalent to those (1.29 × 10^8^ s^−1^ and 1.00 × 10^8^ s^−1^, respectively) in toluene. In contrast, the *k*_nr-solid_ values (4.84 × 10^8^ s^−1^ and 3.38 × 10^8^ s^−1^, respectively) for BBT-2 and BBT-3 in the solid state are larger than those (1.84 × 10^8^ s^−1^ and 1.78 × 10^8^ s^−1^, respectively) in toluene. The *k*_nr-solid_/*k*_r-solid_ values for BBT-2 and BBT-3 in the solid state are 3.55 and 3.76, respectively, which are larger than those (1.43 and 1.78, respectively) in toluene, suggesting that the non-radiative decay in the solid state is accelerated. Consequently, the relatively low *Φ*_fl-solid_ values of BBT-2 and BBT-3 in the solid state are mainly due to the larger *k*_nr-solid_ values compared to those in toluene.

**Table 2:** Photophysical data of 4,4′-bibenzo[*c*]thiophene derivatives in the solid state

Dye	*λ* ^abs-solid^_max_/nm	*λ* ^fl-solid^_max_ a/nm (*Φ*_fl-solid_)	*τ*_fl-solid_ b/ns	*k*_r-solid_ c/s^−1^	*k*_nr-solid_ d/s^−1^	*k*_nr-solid_ */k*_r-solid_
4,4′-BBT (BBT-1)	360	455 (<0.02)	<1	—e	—e	—e
1,1′-Si-4,4′-BBT (BBT-2)	360	435 (0.22)	1.61	1.38 × 10^8^	4.84 × 10^8^	3.55
1,1′,3,3′-Si-4,4′-BBT (BBT-3)	370	435 (0.25)	2.33	9.00 × 10^7^	3.38 × 10^8^	3.76

a Fluorescence quantum yields (*Φ*_fl-solid_) were determined by using a calibrated integrating sphere system (*λ*^ex^ = 360 nm for 4,4′-BBT (BBT-1), 1,1′-Si-4,4′-BBT (BBT-2) and 1,1′,3,3′-Si-4,4′-BBT (BBT-3)).

b Fluorescence lifetime.

c Radiative rate constant (*k*_r-solid_ = *Φ*_fl-solid_/*τ*_fl-solid_).

d Nonradiative rate constant (*k*_nr-solid_ = (1 − *Φ*_fl-solid_)/*τ*_fl-solid_).

e Due to feeble solid-state fluorescence properties.

### Electrochemical properties

**Fig. 5:**
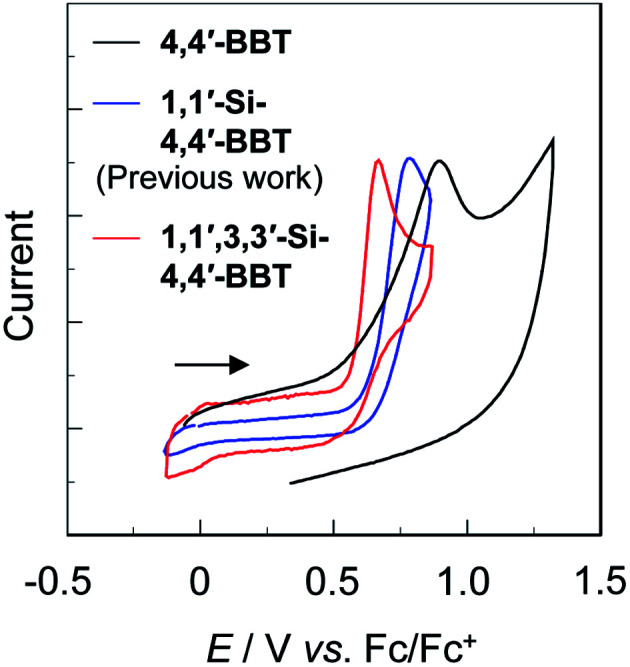
Cyclic voltammograms of 4,4′-BBT (BBT-1), 1,1′-Si-4,4′-BBT (BBT-2) and 1,1′,3,3′-Si-4,4′-BBT (BBT-3) in acetonitrile containing 0.1 M Bu_4_NClO_4_ at a scan rate of 100 mV s^−1^. The arrow denotes the direction of the potential scan.

The electrochemical properties of BBT-1, BBT-2 and BBT-3 were determined using CV in acetonitrile containing 0.1 M tetrabutylammonium perchlorate (Bu_4_NClO_4_). The potentials were internally referenced to ferrocene/ferrocenium (Fc/Fc^+^). The cyclic voltammograms of the three compounds are shown in Fig. 5, and their electrochemical data and the HOMO and LUMO energy levels are summarized in Table 1. For all the three compounds, an irreversible oxidation wave was observed at 0.88 V for BBT-1, 0.78 V for BBT-2 and 0.67 V for BBT-3, while any obvious reduction wave did not appear within the potential window. The oxidation waves for BBT-2 and BBT-3 are cathodically shifted by 0.10 V and 0.21 V, respectively, compared to that for BBT-1, indicating that the introduction of the *tert*-butyldimethylsilyl group into the benzo[*c*]thiophene skeleton can lower the oxidation potential. The HOMO energy levels (−[*E*^ox^_onset_ + 4.8] eV) *versus* vacuum level were estimated from the onset potentials (*E*^ox^_onset_ = 0.75 V for BBT-1, 0.65 V for BBT-2 and 0.54 V for BBT-3) of the oxidation waves, and the LUMO energy levels were estimated from the *E*^ox^_onset_ and intersections (optical energy gap: *E*^opt^_g_ = 3.16 eV for BBT-1, 3.11 eV for BBT-2 and 3.04 eV for BBT-3) of the photoabsorption and fluorescence spectra in toluene. The HOMO energy level rises in the order of BBT-1 (−5.55 eV) < BBT-2 (−5.45 eV) < BBT-3 (−5.34 eV). Similarly, the LUMO energy level rises in the order of BBT-1 (−2.39 eV) < BBT-2 (−2.34 eV) < BBT-3 (−2.30 eV). However, from BBT-1 to BBT-3, the rise of the HOMO energy level is larger than that of the LUMO energy level. Consequently, the fact reveals that the bathochromic shift of the photoabsorption band from BBT-1 to BBT-2 and BBT-3 is mainly attributed to the destabilization of the HOMO energy level through the introduction of the electron-donating *tert*-butyldimethylsilyl group into the benzo[*c*]thiophene skeleton, resulting in a decrease in the HOMO–LUMO band gap.

### Theoretical calculations

**Fig. 6:**
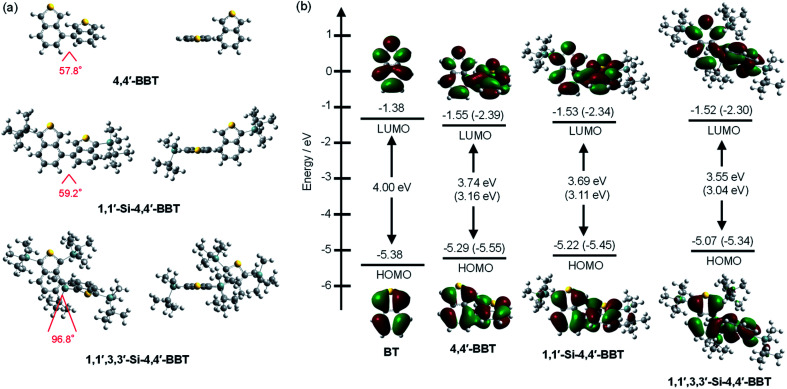
(a) Optimized geometries (top and side views) of 4,4′-BBT (BBT-1), 1,1′-Si-4,4′-BBT (BBT-2) and 1,1′,3,3′-Si-4,4′-BBT (BBT-3) and (b) energy level diagram, HOMO and LUMO of BT and the three 4,4′-bibenzo[*c*]thiophene derivatives derived from DFT calculations at the B3LYP/6-31G(d,p) level. Numbers in parentheses are the experimental values.

**Fig. 7:**
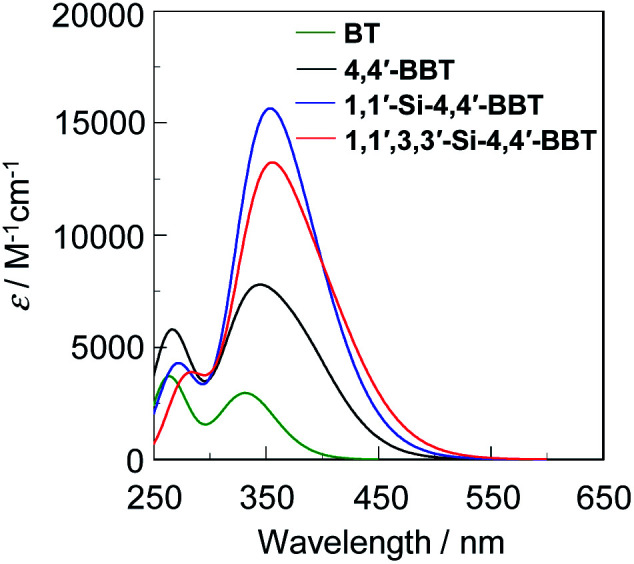
Photoabsorption spectra of BT, 4,4′-BBT (BBT-1), 1,1′-Si-4,4′-BBT (BBT-2) and 1,1′,3,3′-Si-4,4′-BBT (BBT-3) derived from TD-DFT calculations.

In order to examine the electronic structures of the 4,4′-bibenzo[*c*]thiophene derivatives, the molecular structures and molecular orbitals of BBT-1, BBT-2 and BBT-3 and benzo[*c*]thiophene (BT) as a reference were calculated using DFT at the B3LYP/6-31G(d,p) level^22^ (Fig. 6). The DFT calculations demonstrate that the calculated dihedral angles between the two benzo[*c*]thiophene units are 57.8° for BBT-1, 59.2° for BBT-2 and 96.8° for BBT-3, that is, the two units in BBT-3 twist considerably due to the *tert*-butyldimethylsilyl groups at the 3,3′-positions, compared to those in BBT-1 and BBT-2 (Fig. 6a). Therefore, good correlation was observed between the molecular structures estimated by the DFT calculations and experimentally obtained from the X-ray crystal structure analysis, although for BBT-3, the calculated dihedral angle between the two benzo[*c*]thiophene units is larger than that determined from the X-ray crystal structure analysis (Fig. 3a and b). As shown in Fig. 6b, for the three 4,4′-bibenzo[*c*]thiophene derivatives the HOMO are delocalized on each benzo[*c*]thiophene unit, as with the pattern of BT. However, the LUMO for the three 4,4′-bibenzo[*c*]thiophene derivatives are delocalized over the whole molecule through the 4,4′-positions. It was found that the HOMO energy levels of the three 4,4′-bibenzo[*c*]thiophene derivatives are higher than that (−5.38 eV) of BT, but their LUMO energy levels are lower than that (−1.38 eV) of BT. The HOMO and LUMO energy levels rise in the order of BBT-1 (−5.29 eV and −1.55 eV) < BBT-2 (−5.22 eV and −1.53 eV) < BBT-3 (−5.07 eV and −1.52 eV), while from BBT-1 to BBT-3, the rise of the HOMO energy level is larger than that of the LUMO energy level, resulting in a decrease in the HOMO–LUMO band gap. Moreover, the time-dependent density functional theory (TD-DFT) calculations indicate that the calculated *λ*^abs-calcd^_max_ of BBT-1, BBT-2 and BBT-3 is 345 nm, 354 nm and 356 nm, respectively, which appears in a significantly longer wavelength region than that (332 nm) of BT (Fig. 7). For BT and the three 4,4′-bibenzo[*c*]thiophene derivative, the S_0_ → S_1_ transitions are mainly attributed to the transitions from the HOMO to the LUMO (70% for BT, 67% for BBT-1, 67% for BBT-2 and 68% for BBT-3). The corresponding oscillator strength (*f*) value increases in the order of BT (0.07) < BBT-1 (0.11) < BBT-3 (0.14) < BBT-2 (0.16), and indeed, the calculated *ε*_calcd_ value also increases in the order of BT (3000 M^−1^ cm^−1^) < BBT-1 (7800 M^−1^ cm^−1^) < BBT-3 (13 200 M^−1^ cm^−1^) < BBT-2 (15 600 M^−1^ cm^−1^). Thus, the DFT calculations reveal that the bathochromic shift of the photoabsorption band from BBT-1 to BBT-2 and BBT-3 is ascribable to the destabilization of the HOMO energy level through the introduction of the electron-donating *tert*-butyldimethylsilyl group into the benzo[*c*]thiophene skeleton. Consequently, the DFT calculations are in good agreement with the experimental results from the CV and the photoabsorption and fluorescence spectral analyses, although the *f* and *ε*_calcd_ values of BBT-3 are slightly lower than those of BBT-2, which is opposite in the values to the experimental results.

## Conclusions

We have achieved a facile synthesis of 4,4′-bibenzo[*c*]thiophene derivatives, unsubstituted 4,4′-bibenzo[*c*]thiophene 4,4′-BBT and its silyl-substituted derivatives 1,1′-Si-4,4′-BBT and 1,1′,3,3′-Si-4,4′-BBT with one or two *tert*-butyldimethylsilyl groups on each thiophene ring, and revealed their photophysical properties in the solution and in the solid state and electrochemical properties. It was found that the bathochromic shift of the photoabsorption band from 4,4′-BBT to 1,1′-Si-4,4′-BBT and 1,1′,3,3′-Si-4,4′-BBT is mainly attributed to the destabilization of the HOMO energy level through the introduction of the electron-donating *tert*-butyldimethylsilyl group into the benzo[*c*]thiophene skeleton, resulting in a decrease in the HOMO–LUMO band gap. The three 4,4′-bibenzo[*c*]thiophene derivatives exhibit moderate fluorescence properties in the solution (*Φ*_fl_ = *ca.* 0.4). Moreover, the silyl-substituted derivatives 1,1′-Si-4,4′-BBT and 1,1′,3,3′-Si-4,4′-BBT show relatively high *Φ*_fl-solid_ value (0.22 and 0.25, respectively) in the solid state, compared to 4,4′-BBT (*Φ*_fl-solid_ < 0.02). The DFT calculations demonstrate that the HOMO energy levels of the 4,4′-bibenzo[*c*]thiophene derivatives are higher than that of benzo[*c*]thiophene (BT), while their LUMO energy levels are lower than that of BT. Consequently, this work opened up a new way for not only synthetic method and photophysical and electrochemical properties of 4,4′-bibenzo[*c*]thiophene derivatives with the different substituents on the thiophene rings, but also development of fused-bibenzo[*c*]thiophene derivatives as functional dyes.

## Experimental

### General

Melting points were measured with an AS ONE ATM-02. IR spectra were recorded on a SHIMADZU IRTracer-100 by ATR method. ^1^H NMR and ^13^C NMR spectra were recorded on a Varian-500 FT NMR spectrometer and NMR solvent was used as an external standard for calibration. High-resolution mass spectral data by APCI and GC-EI were acquired on a Thermo Fisher Scientific LTQ Orbitrap XL and JEOL JMS-T100 GCV 4G, respectively. Recycling gel permeation chromatography (GPC) were preformed using RI-detector (GL Science RI 704) and pump (GILSON 307 PUMP) with column (Shodex GPC H-2001L). Photoabsorption spectra of solution were observed with a Shimadzu UV-3600 plus spectrophotometer. Photoabsorption spectra of the solid were recorded by a Shimadzu UV-3600 plus spectrophotometer with a calibrated integrating sphere system. Fluorescence spectra of solution and the solid were measured with a HORIBA FluoroMax-4 spectrofluorometer. The fluorescence quantum yields in solution and in the solid state were determined using a HORIBA FluoroMax-4 spectrofluorometer with a calibrated integrating sphere system. Fluorescence decay measurements were performed on a HORIBA DeltaFlex modular fluorescence lifetime system, using a Nano LED pulsed diode excitation source (370 nm). Cyclic voltammetry (CV) curves were recorded in acetonitrile/Bu_4_NClO_4_ (0.1 M) solution at a scan rate of 100 mV s^−1^ with a three-electrode system consisting of Ag/Ag^+^ as the reference electrode, a Pt plate as the working electrode and a Pt wire as the counter electrode using an Electrochemical Measurement System HZ-7000 (HOKUTO DENKO).

### Synthesis

#### 4,4′-Bibenzo[*c*]thiophene (4,4′-BBT)

To a THF solution (20 mL) of 1,1′,3,3′-tetrahydro-[4,4′-bibenzo[*c*]thiophene] 2,2′-dioxide (1)^20^ (0.20 g, 0.66 mmol) under a nitrogen atmosphere at 0 °C was added dropwise a 1.3 M THF solution of lithium hexamethyldisilazide (3.1 mL, 4.0 mmol). After stirring for 3 h, the reaction mixture was quenched with water, and then, the solution was extracted with ethyl acetate. The ethyl acetate extract was dried over anhydrous MgSO_4_, filtrated and concentrated. Recycling GPC (toluene as eluent) was performed to give 4,4′-BBT (0.09 g, yield 51%) as a light-yellow solid; decomposed at around 145 °C; FT-IR (ATR): *ν̃* = 3103, 1686, 1172, 864 cm^−1^; ^1^H NMR (500 MHz, acetone-*d*_6_): *δ* = 7.19–7.24 (m, 4H), 7.59 (dd, *J* = 1.1 and 3.4 Hz, 2H), 7.75 (dt, 2H), 8.00 (d, *J* = 3.4 Hz, 2H) ppm; ^13^C NMR (125 MHz, acetone-*d*_6_): *δ* = 117.96, 118.31, 122.51, 124.29, 124.35, 134.56, 138.45, 139.91 ppm; HRMS (GC-EI): *m*/*z* (%): [M − H^+^] calcd for C_16_H_9_S_2_, 265.01457; found 265.01442.

#### 1,1′-Bis(*tert*-butyldimethylsilyl)-4,4′-bibenzo[*c*]thiophene (1,1′-Si-4,4′-BBT)

(Method A: previous work)^20^ To a THF solution (60 mL) of 1 (0.50 g, 1.65 mmol) under an argon atmosphere at −80 °C was added tetramethylethylenediamine (1.48 g, 9.92 mmol). After stirring for 30 min, a 1.6 M hexane solution of *n*BuLi (6.20 mL, 9.92 mmol) was added dropwise for 30 min, and then, a THF solution (10 mL) of *tert*-butyldimethylsilyl chloride (0.75 g, 4.96 mmol) was added dropwise for 20 min. After stirring for 12 h at room temperature, the reaction mixture was quenched with water, and then, the solution was extracted with ethyl acetate. The ethyl acetate extract was dried over anhydrous MgSO_4_, filtrated and concentrated. The residue was chromatographed on silica gel (hexane as eluent), and then, recycling GPC (toluene as eluent) was performed to give 1,1′-Si-4,4′-BBT (0.33 g, yield 40%) as a light-yellow solid. (Method B) To a THF solution (0.8 mL) of 4,4′-BBT (0.14 g, 0.53 mmol) under a nitrogen atmosphere at 0 °C was added dropwise a 1.0 M hexane/THF solution of lithium diisopropylamide (1.6 mL, 1.6 mmol). After stirring for 3 h, a THF solution (0.3 mL) of *tert*-butyldimethylsilyl chloride (0.24 g, 1.6 mmol) was added dropwise. The reaction mixture was further stirred for 12 h. The reaction mixture was quenched with water, and then, the solution was extracted with ethyl acetate. The ethyl acetate extract was dried over anhydrous MgSO_4_, filtrated and concentrated. The residue was chromatographed on silica gel (dichloromethane : hexane = 1 : 1 as eluent) and then on alumina (dichloromethane : hexane = 1 : 3 as eluent) to give 1,1′-Si-4,4′-BBT (0.095 g, yield 36%) as a yellow solid; mp 153–155 °C; FT-IR (ATR): *ν̃* = 2949 (aliphatic C–H str.), 2926 (aliphatic C–H str.), 2855 (aliphatic C–H str.), 1458 (Si–C(Ar) str.), 1360, 1250 (aliphatic Si–CH_3_ str.), 804 (aliphatic Si–CH_3_ str.) cm^−1^; ^1^H NMR (500 MHz, CDCl_3_): *δ* = 0.53 (s, 12H), 0.99 (s, 18H), 7.18–7.23 (m, 4H), 7.78 (d, *J* = 8.1 Hz, 2H), 7.81 (s, 2H) ppm; ^13^C NMR (125 MHz, CDCl_3_): *δ* = −3.73, 18.37, 26.92, 123.17, 123.25, 123.50, 124.03, 128.34, 134.88, 140.20, 145.12 ppm; HRMS (APCI): *m*/*z* (%): [M + H^+^] calcd for C_28_H_39_S_2_Si_2_, 495.20262; found 495.20303.

#### 1,1′,3,3′-Tetrakis(*tert*-butyldimethylsilyl)-4,4′-bibenzo[*c*]thiophene (1,1′,3,3′-Si-4,4′-BBT)

(Method A) To a THF solution (0.8 mL) of 4,4′-BBT (0.14 g, 0.53 mmol) under a nitrogen atmosphere at 0 °C was added dropwise a 1.0 M hexane/THF solution of lithium diisopropylamide (4.2 mL, 4.2 mmol). After stirring for 3 h, a THF solution (1.0 mL) of *tert*-butyldimethylsilyl chloride (0.62 g, 4.2 mmol) was added dropwise. The reaction mixture was further stirred for 12 h. The reaction mixture was quenched with water, and then, the solution was extracted with ethyl acetate. The ethyl acetate extract was dried over anhydrous MgSO_4_, filtrated and concentrated. The residue was chromatographed on silica gel (dichloromethane : hexane = 1 : 3 as eluent) to give 1,1′,3,3′-Si-4,4′-BBT (0.1 g, yield 26%) as a white solid. (Method B) To a THF solution (0.3 mL) of 1,1′-Si-4,4′-BBT (0.07 g, 0.14 mmol) under a nitrogen atmosphere at 0 °C was added dropwise a 1.0 M hexane/THF solution of lithium diisopropylamide (1.4 mL, 1.4 mmol). After stirring for 3 h, a THF solution (0.3 mL) of *tert*-butyldimethylsilyl chloride (0.21 g, 1.4 mmol) was added dropwise. The reaction mixture was further stirred for 14 h. The reaction mixture was quenched with water, and then, the solution was extracted with dichloromethane. The dichloromethane extract was dried over anhydrous MgSO_4_, filtrated and concentrated. The residue was chromatographed on silica gel (hexane as eluent) to give 1,1′,3,3′-Si-4,4′-BBT (0.04 g, yield 40%) as a white solid; mp 172–173 °C; FT-IR (ATR): *ν̃* = 2955 (aliphatic C–H str.), 2926 (aliphatic C–H str.), 2855 (aliphatic C–H str.), 1470 (Si–C(Ar) str.), 1362, 1250 (aliphatic Si–CH_3_ str.), 804 (aliphatic Si–CH_3_ str.) cm^−1^; ^1^H NMR (500 MHz, acetone-*d*_6_): *δ* = −0.87 (s, 3H), −0.04 (s, 3H), 0.57 (s, 3H), 0.62 (s, 3H), 0.77 (s, 9H), 0.99 (s, 9H), 7.02 (dd, *J* = 1.2 and 6.6 Hz, 2H), 7.19–7.22 (m, 2H), 7.99 (dd, *J* = 8.8 Hz, 2H) ppm; ^13^C NMR (125 MHz, acetone-*d*_6_): *δ* = −3.78, −3.61, 18.97, 19.32, 27.15, 28.25, 123.12, 125.27, 128.83, 136.01, 136.12, 139.28, 147.78, 149.76 ppm; HRMS (APCI): *m*/*z* (%): [M^+•^] calcd for C_40_H_66_S_2_Si_4_, 722.36775; found 722.36843.

### X-ray crystallographic analysis

The reflection data were collected at 100 K on a Bruker AXS SMART APEX II ULTRA diffractometer using monochromated Mo-Kα (*λ* = 0.71073 Å). The structure was solved by the SHELXT 2014/5 method and refined based on full-matrix least squares on *F*^2^ using SHELXL-2017/1. The non-hydrogen atoms were refined anisotropically. Hydrogen atoms were fixed geometrically and not refined. Crystallographic data have been deposited in the Cambridge Crystallographic Data Centre (CCDC 2055734† for 1,1′-Si-4,4′-BBT and CCDC 2055735† for 1,1′,3,3′-Si-4,4′-BBT, respectively).

#### Crystal of 1,1′-Si-4,4′-BBT

A suitable single crystal of 1,1′-Si-4,4′-BBT was grown by slow evaporation of acetone/ethanol solution at room temperature for several days, as colorless block crystal, air stable. Crystallographic data: C_28_H_38_S_2_Si_2_, *M* = 494.88, monoclinic, *a* = 17.6427(9), *b* = 11.2395(6), *c* = 14.1794(7) Å, *β* = 95.121(1)°, *V* = 2800.5(2) Å^3^, *D*_calcd_ = 1.174 g cm^−3^, space group *P*2_1_/*c* (no. 14), *Z* = 4, 17 974 reflections measured, 6739 unique (*R*_int_ = 0.027), which were used in all calculations. The final *R*_1_(reflections) = 0.0292(5949) [*I* > 2*σ*(*I*)], w*R*_2_(reflections) = 0.0743(6739). GOF = 0.955 (Table S1†).

#### Crystal of 1,1′,3,3′-Si-4,4′-BBT

A suitable single crystal of 1,1′,3,3′-Si-4,4′-BBT was grown by slow evaporation of acetone/ethanol solution at room temperature for several days, as colorless plate crystal, air stable. Crystallographic data: C_40_H_66_S_2_Si_4_, *M* = 723.40, triclinic, *a* = 7.5572(9), *b* = 13.6372(16), *c* = 22.295(3) Å, *α* = 72.5230(10)°, *β* = 89.9300(10)°, *γ* = 81.0900(10)°, *V* = 2162.7(4) Å^3^, *D*_calcd_ = 1.111 g cm^−3^, space group *P*1̄ (no. 2), *Z* = 2, 10 428 reflections measured, 10 234 unique (*R*_int_ = 0.041), which were used in all calculations. The final *R*_1_(reflections) = 0.0449(7519) [*I* > 2*σ*(*I*)], w*R*_2_(reflections) = 0.1092(10 234). GOF = 1.011 (Table S1†).

## Conflicts of interest

There are no conflicts to declare.

## Supplementary Material

RA-011-D1RA01189H-s001

RA-011-D1RA01189H-s002
