# Supplementary material for: Synthesis, optical and electrochemical properties of 4,4′-bibenzo[c]thiophene derivatives
Source: RSC Adv. 2021 May 25;11(31):18870–80. doi: 10.1039/d1ra01189h (PMC9033462; doi:10.1039/d1ra01189h)
Supplement: RA-011-D1RA01189H-s001 [file RA-011-D1RA01189H-s001.pdf]

Electronic Supplementary Material (ESI) for RSC Advances.
This journal is © The Royal Society of Chemistry 2021

Supplementary Information

Synthesis, optical and electrochemical properties of 4,4'-bibenzo[*c*]thiophene derivatives

Kotaro Obayashi,[a] Keiichi Imato,[a] Satoshi Aoyama,[a] Toshiaki Enoki,[a] Seiji Akiyama,[b] Mio Ishida,[b] Seiji Suga,[c] Koichi Mitsudo[c] and Yousuke Ooyama*[a]

[a]*Department of Applied Chemistry, Graduate School of Engineering, Hiroshima University, 1-4-1 Kagamiyama, Higashi-Hiroshima 739-8527, Japan. E-mail: yooyama@hiroshima-u.ac.jp; Fax: (+81) 82-424-5494*

[b]*Science & Innovation Center, Mitsubishi Chemical Corporation, 1000, Kamoshida-cho, Aoba-ku, Yokohama-shi, Kanagawa 227-8502, Japan*

[c]*Division of Applied Chemistry, Graduate School of Natural Science and Technology, Okayama University, 3-1-1 Tsushima-naka, Kita-ku, Okayama 700-8530, Japan*

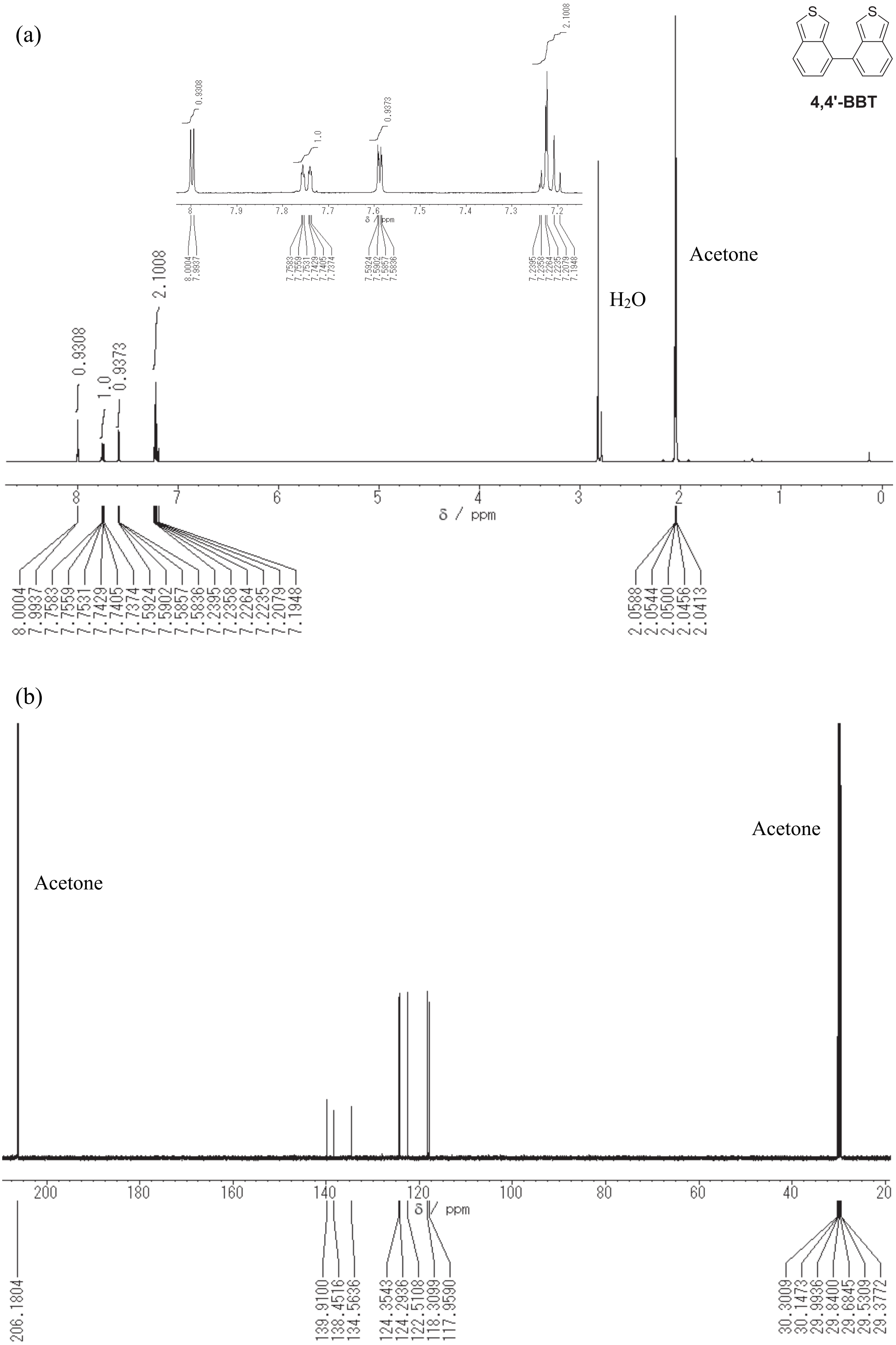

Fig. S1 (a) ^{1}H NMR (500 MHz) and (b) ^{13}C NMR (125 MHz) spectra of **4,4′-BBT** in acetone-d_6.

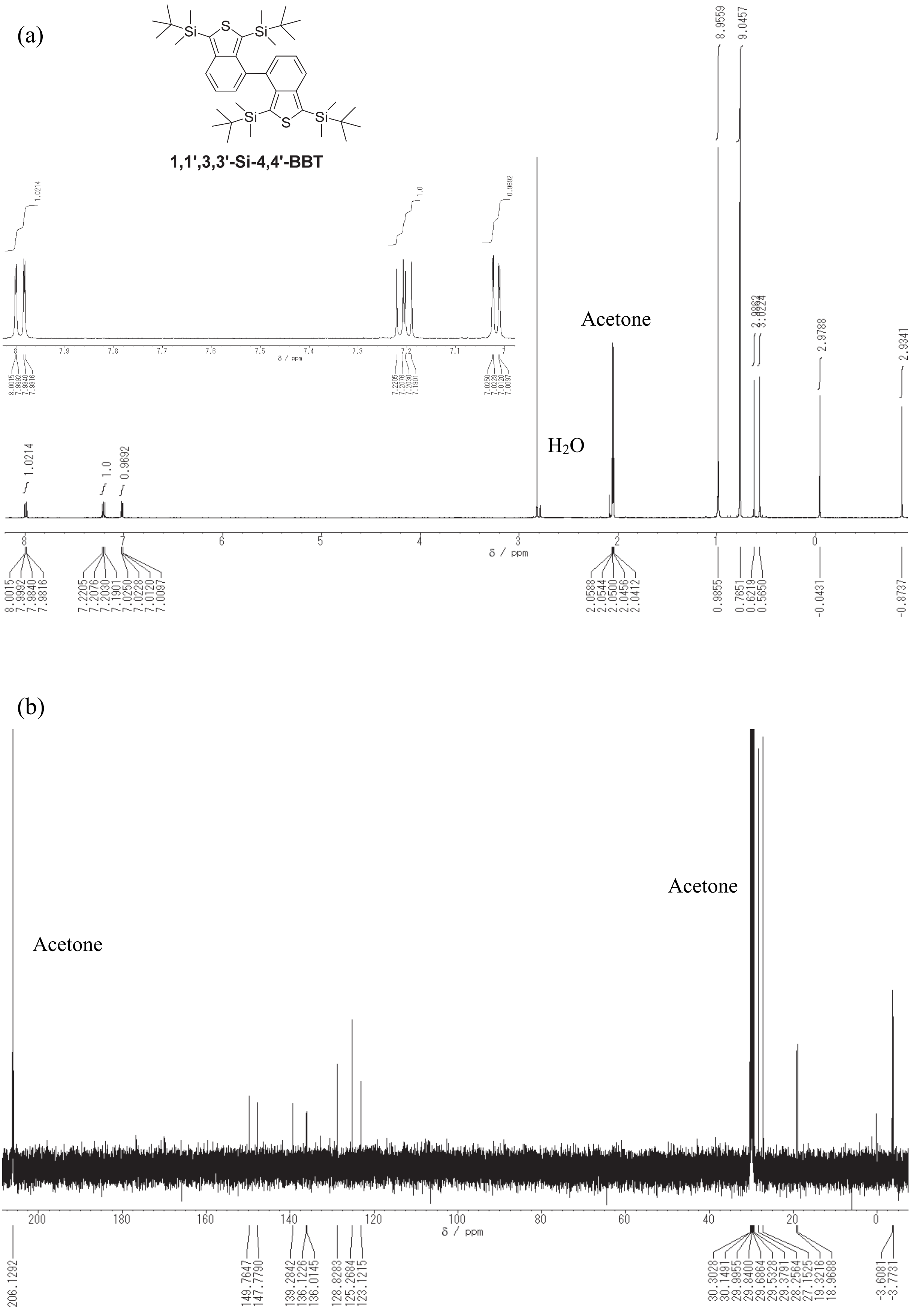

Fig. S2 (a) ^{1}H NMR (500 MHz) and (b) ^{13}C NMR (125 MHz) spectra of **1,1′,3,3′-Si-4,4′-BBT** in acetone-d_6.

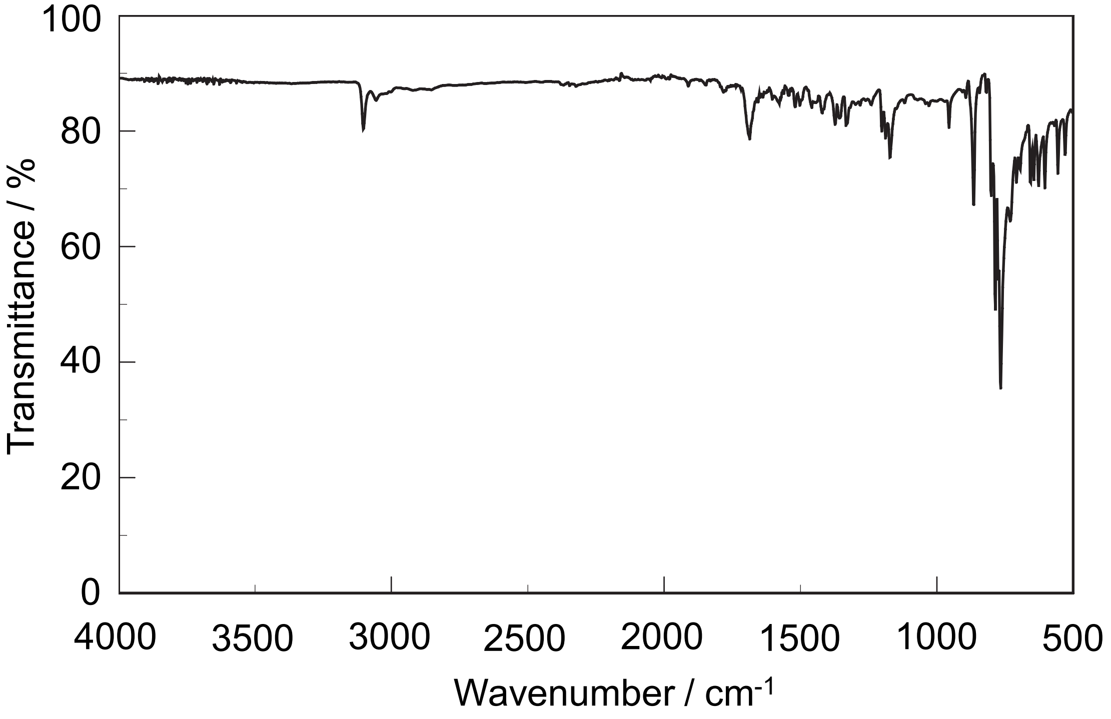

Fig. S3 FTIR spectrum of **4,4′-BBT**.

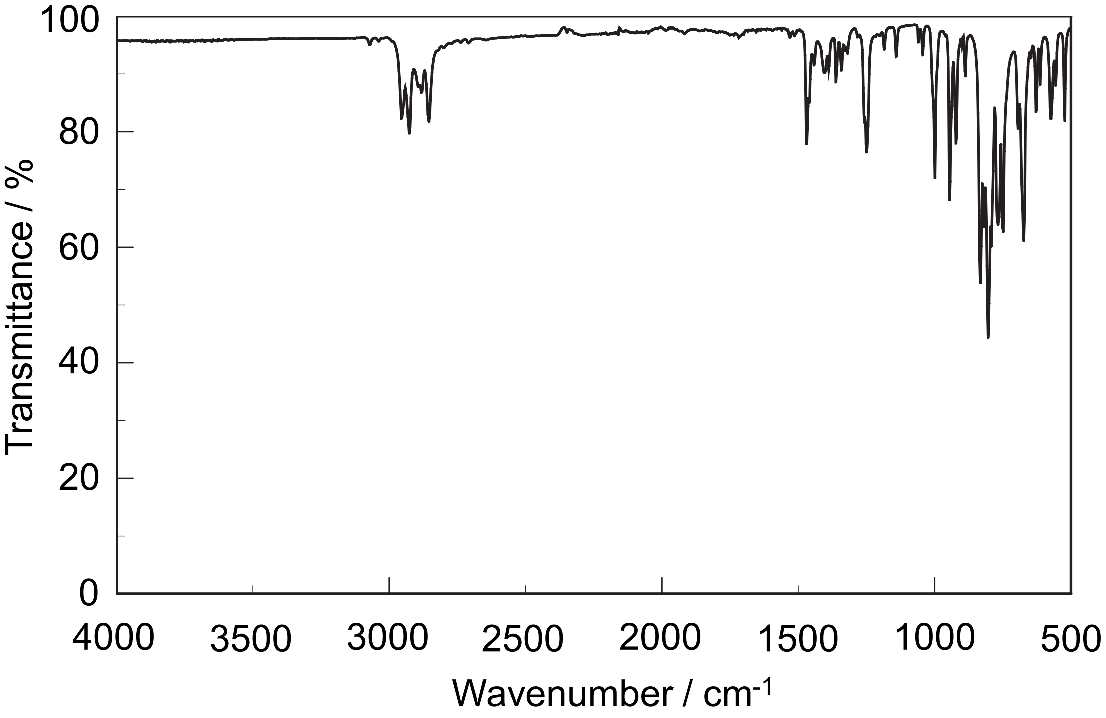

Fig. S4 FTIR spectrum of **1,1′,3,3′-Si-4,4′-BBT**.

Table S1 Crystal data and structure refinement parameters for **1,1′-Si-4,4′-BBT (**CCDC 2055734) and **1,1′,3,3′-Si-4,4′-BBT** (CCDC 2055735).

Compound	**1,1′-Si-4,4′-BBT**	**1,1′,3,3′-Si-4,4′-BBT**
Molecular formula	$C_{28}H_{38}S_2Si_2$	$C_{40}H_{66}S_2Si_4$
Formula weight	494.88	723.40
Number of reflection used for unit cell determination (2θ range/°)	17974 (4.22-57.8)	10428 (4.17-57.5)
Temperature/K	100(2)	100(2)
Crystal System	monoclinic	triclinic
Space group	$P2_1/c$	P1-
a/Å	17.6427(9)	7.5572(9)
b/Å	11.2395(6)	13.6372(16)
c/Å	4.1794(7)	22.295(3)
α/°		72.523(1)
β/°	95.121(1)	89.930(1)
γ/°		81.090(1)
V/Å^3	2800.5(2)	2162.7(4)
Z	4	2
D_c/g cm^{-3}	1.174	1.111
F(000)	1064.0	788.00
Radiation	Mo-Kα (λ = 0.71073 Å)	Mo-Kα (λ = 0.71073 Å)
Crystal size/mm^3	0.22×0.16×0.11	0.15×0.11×0.06
Range of induces *h*; *k*; *l*	-23, 23; -14, 14; -18, 18	-9, 9; -17, 17; -29, 29
Reflections collected (unique)	6739	10234
Reflection observed with $I_0>2\sigma I_0$	5949	7519
Number of parameters	299	435
Final R indexes [$I_0>2\sigma I_0$]	R_1 = 0.0292, wR_2 ＝0.0711	R_1 = 0.0449, wR_2 ＝0.0991
Final R indexes [all data]	R_1 = 0.0343, wR_2 ＝0.0743	R_1 = 0.0681, wR_2 ＝0.1092
Goodness-of-fit on F^2	0.955	1.011
Max. Shift/Error in final cycle	0.00	0.00
Max. peak in final diff. map/e Å^{-3}	0.43	0.580
Min. peak in final diff. map/e Å^{-3}	-0.205	-0.418

Table S2 Geometrical coordinates of the optimized **BT** by DFT at the B3LYP/6-31G(d,p) level.

Cartesian coordinates:

Center Number	Atomic Number	Atomic Type	Coordinates (Angstroms) X	Y	Z
1	C	0	5.424758	-0.264737	6.75859
2	C	0	5.82839	1.829788	5.626265
3	C	0	4.460674	1.882935	5.210211
4	C	0	3.577831	0.894402	5.544278
5	C	0	4.032786	-0.205875	6.336334
6	S	0	4.266367	-2.334082	7.755654
7	H	0	6.498162	2.62934	5.324315
8	H	0	4.131108	2.727258	4.611905
9	H	0	2.540761	0.934689	5.224789
10	C	0	5.677591	-1.377122	7.546215
11	H	0	6.609643	-1.674254	8.002156
12	C	0	3.295851	-1.280127	6.808395
13	H	0	2.247253	-1.48983	6.653107
14	C	0	6.333896	0.790644	6.372756
15	H	0	7.370667	0.802544	6.637076

Table S3 Geometrical coordinates of the optimized **4,4′-BBT** by DFT at the B3LYP/6-31G(d,p) level.

Cartesian coordinates:

Center Number	Atomic Number	Atomic Type	Coordinates (Angstroms) X	Y	Z
1	C	0	3.014613	0.092656	0.246587
2	C	0	3.469568	1.192933	1.038642
3	C	0	2.586725	2.181467	1.372708
4	C	0	1.21901	2.12832	0.956653
5	C	0	0.713504	1.089175	0.210163
6	C	0	1.622641	0.033794	-0.17567
7	S	0	2.781034	-2.035549	-1.172737
8	C	0	-1.622642	0.033794	0.17567
9	C	0	-0.713504	1.089175	-0.210163
10	C	0	-1.21901	2.128319	-0.956655
11	C	0	-2.586726	2.181466	-1.372709
12	C	0	-3.469569	1.192933	-1.038641
13	C	0	-3.014613	0.092656	-0.246585

14	S	0	-2.781033	-2.035551	1.172734
15	H	0	4.506637	1.23322	1.358132
16	H	0	2.916292	3.02579	1.971013
17	H	0	0.549238	2.927872	1.258602
18	H	0	-0.549238	2.927871	-1.258604
19	H	0	-2.916292	3.025789	-1.971014
20	H	0	-4.506638	1.23322	-1.35813
21	C	0	3.751548	-0.981598	-0.225472
22	H	0	4.800145	-1.191301	-0.070182
23	C	0	1.369808	-1.078593	-0.963293
24	H	0	0.437756	-1.375725	-1.419233
25	C	0	-1.369809	-1.078591	0.963295
26	H	0	-0.437757	-1.375723	1.419236
27	C	0	-3.751549	-0.981596	0.225475
28	H	0	-4.800146	-1.191299	0.070187

Table S4 Geometrical coordinates of the optimized **1,1′-Si-4,4′-BBT** by DFT at the B3LYP/6-31G(d,p) level.

Cartesian coordinates:

Center	Atomic	Atomic	Coordinates	(Angstroms)	
Number	Number	Type	X	Y	Z
1	C	0	-3.788283	-0.492598	0.089297
2	6	0	-2.986115	0.584641	0.498518
3	6	0	-3.354889	1.679423	1.342929
4	1	0	-4.353832	1.726187	1.760648
5	6	0	-2.446402	2.667101	1.610884
6	1	0	-2.726166	3.500481	2.248781
7	6	0	-1.123347	2.626604	1.076345
8	1	0	-0.429175	3.423266	1.326347
9	6	0	-0.691749	1.595562	0.27455
10	6	0	-1.633951	0.547765	-0.040376
11	6	0	-1.439422	-0.550958	-0.863946
12	1	0	-0.544149	-0.828669	-1.400053
13	6	0	3.788279	-0.492564	-0.089025
14	6	0	2.986111	0.584665	-0.498303
15	6	0	3.354833	1.679343	-1.342865
16	1	0	4.353769	1.726105	-1.760593

17	6	0	2.446313	2.666963	-1.610939
18	1	0	2.726049	3.500266	-2.248949
19	6	0	1.123272	2.6265	-1.076373
20	1	0	0.429071	3.423114	-1.326459
21	6	0	0.691724	1.595561	-0.274423
22	6	0	1.633958	0.54783	0.040635
23	6	0	1.439487	-0.550772	0.864378
24	1	0	0.544258	-0.828431	1.40058
25	6	0	-5.775448	-2.80348	0.117712
26	1	0	-5.115385	-3.388372	0.766622
27	1	0	-5.541246	-3.069003	-0.91798
28	1	0	-6.802794	-3.127467	0.315495
29	6	0	-5.961292	-0.662265	2.29951
30	1	0	-5.187012	-1.117294	2.925481
31	1	0	-6.917186	-1.127968	2.563458
32	1	0	-6.027412	0.395254	2.570815
33	6	0	-6.799976	0.05814	-0.646783
34	6	0	-6.528867	-0.243837	-2.135594
35	1	0	-7.215503	0.331075	-2.771978
36	1	0	-6.676543	-1.302979	-2.374201
37	1	0	-5.508115	0.025791	-2.426611
38	6	0	-6.628465	1.57261	-0.407848
39	1	0	-7.322148	2.13865	-1.044704
40	1	0	-5.61479	1.91176	-0.644004
41	1	0	-6.844201	1.85281	0.629736
42	6	0	-8.25415	-0.338967	-0.309643
43	1	0	-8.509347	-0.122558	0.733774
44	1	0	-8.443987	-1.403329	-0.48752
45	1	0	-8.957057	0.224301	-0.938629
46	6	0	5.775378	-2.80351	-0.117233
47	1	0	5.541004	-3.06881	0.918478
48	1	0	6.802756	-3.127533	-0.314775
49	1	0	5.115422	-3.388554	-0.76612
50	6	0	5.960738	-0.662927	-2.299662
51	1	0	6.916704	-1.128404	-2.563741
52	1	0	6.026395	0.394508	-2.571388
53	1	0	5.18641	-1.118454	-2.925217
54	6	0	6.800145	0.058268	0.646325
55	6	0	6.528735	-0.242702	2.135287

56	1	0	7.215662	0.332164	2.771399
57	1	0	6.675754	-1.301796	2.374515
58	1	0	5.508118	0.0277	2.426048
59	6	0	8.254212	-0.339586	0.309616
60	1	0	8.509571	-0.124122	-0.733961
61	1	0	8.443676	-1.403859	0.488388
62	1	0	8.957259	0.223973	0.938188
63	6	0	6.629232	1.572656	0.406444
64	1	0	5.615648	1.912337	0.642237
65	1	0	6.845255	1.852135	-0.631275
66	1	0	7.32301	2.138827	1.043079
67	16	0	-2.849542	-1.51168	-0.958291
68	16	0	2.849604	-1.511491	0.958738
69	14	0	-5.583553	-0.949556	0.463523
70	14	0	5.583476	-0.949669	-0.463492

Table S5 Geometrical coordinates of the optimized **1,1′,3,3′-Si-4,4′-BBT** by DFT at the B3LYP/6-31G(d,p) level.

Cartesian coordinates:

Center	Atomic	Atomic	Coordinates	(Angstroms)	
Number	Number	Type	X	Y	Z
1	C	0	4.218161	-0.33767	-0.016252
2	C	0	3.066512	-0.77509	0.653943
3	C	0	3.009221	-1.909125	1.51953
4	H	0	3.910183	-2.480712	1.707261
5	C	0	1.826697	-2.26251	2.105088
6	H	0	1.773574	-3.118151	2.771738
7	C	0	0.656908	-1.493972	1.862646
8	H	0	-0.261931	-1.769139	2.369861
9	C	0	0.637633	-0.390744	1.036687
10	C	0	1.866232	0.024955	0.386908
11	C	0	2.114446	1.088983	-0.514497
12	C	0	6.809855	-0.307254	-1.620217
13	H	0	6.802518	0.785533	-1.678026
14	H	0	7.853335	-0.633829	-1.684102
15	H	0	6.290831	-0.688549	-2.505699
16	C	0	6.041037	-2.86158	-0.122845

17	H	0	5.34186	-3.226144	-0.882163
18	H	0	7.04384	-3.200161	-0.405932
19	H	0	5.787633	-3.348789	0.823057
20	C	0	6.970751	-0.33548	1.509924
21	C	0	8.420041	-0.867745	1.459713
22	H	0	8.985098	-0.512927	2.332474
23	H	0	8.457503	-1.962788	1.473604
24	H	0	8.955707	-0.525105	0.56745
25	C	0	6.303838	-0.827547	2.811122
26	H	0	5.273994	-0.468341	2.904417
27	H	0	6.285945	-1.921581	2.876675
28	H	0	6.860867	-0.462198	3.684818
29	C	0	7.001599	1.207344	1.520032
30	H	0	7.549511	1.571454	2.399883
31	H	0	7.502412	1.61494	0.63493
32	H	0	5.993996	1.634556	1.560787
33	C	0	1.916458	2.515077	-3.191008
34	H	0	1.474095	3.334924	-3.768268
35	H	0	3.003072	2.617719	-3.251161
36	H	0	1.646205	1.579186	-3.691998
37	C	0	-0.639894	2.357931	-1.619016
38	H	0	-0.921469	1.343743	-1.912766
39	H	0	-1.205427	2.61625	-0.722451
40	H	0	-0.95275	3.034042	-2.422946
41	C	0	1.658739	4.242796	-0.610874
42	C	0	3.144123	4.60451	-0.82185
43	H	0	3.816352	3.883698	-0.345652
44	H	0	3.410277	4.660591	-1.882733
45	H	0	3.356494	5.588106	-0.380499
46	C	0	1.363225	4.252281	0.901551
47	H	0	1.603609	5.235979	1.328893
48	H	0	0.308586	4.051797	1.113043
49	H	0	1.961625	3.50643	1.435645
50	C	0	0.792869	5.329558	-1.289518
51	H	0	1.03961	6.318106	-0.878443
52	H	0	0.962508	5.379704	-2.371199
53	H	0	-0.277122	5.166474	-1.124974
54	C	0	-4.218199	0.337696	-0.01623
55	C	0	-3.066543	0.775158	0.653922

56	C	0	-3.009278	1.90918	1.519525
57	H	0	-3.910269	2.4807	1.70732
58	C	0	-1.826754	2.262597	2.105064
59	H	0	-1.773638	3.118237	2.771718
60	C	0	-0.656959	1.49407	1.862627
61	H	0	0.261867	1.769224	2.369871
62	C	0	-0.63765	0.390855	1.036651
63	C	0	-1.866239	-0.024847	0.386848
64	C	0	-2.11446	-1.088901	-0.51452
65	C	0	-6.809806	0.307577	-1.620348
66	H	0	-6.802379	-0.785193	-1.678443
67	H	0	-7.853311	0.634084	-1.684188
68	H	0	-6.290778	0.689149	-2.505709
69	C	0	-6.041244	2.861592	-0.122269
70	H	0	-5.34176	3.226545	-0.881112
71	H	0	-7.043971	3.200087	-0.405734
72	H	0	-5.788418	3.348522	0.823933
73	C	0	-6.970791	0.335081	1.509822
74	C	0	-6.303554	0.826421	2.811127
75	H	0	-5.273783	0.466915	2.904103
76	H	0	-6.28536	1.920421	2.877146
77	H	0	-6.860539	0.460843	3.684755
78	C	0	-8.419941	0.867762	1.460035
79	H	0	-8.45709	1.962805	1.474463
80	H	0	-8.955802	0.525708	0.567662
81	H	0	-8.985003	0.51267	2.332681
82	C	0	-7.002046	-1.207739	1.519275
83	H	0	-7.549808	-1.572088	2.39912
84	H	0	-7.503215	-1.614811	0.634134
85	H	0	-5.994546	-1.635247	1.559566
86	C	0	-1.91645	-2.51493	-3.191073
87	H	0	-1.47409	-3.334782	-3.768334
88	H	0	-3.003066	-2.617585	-3.251208
89	H	0	-1.646206	-1.579043	-3.692072
90	C	0	0.639921	-2.357663	-1.619065
91	H	0	0.921517	-1.343309	-1.912209
92	H	0	1.205474	-2.616495	-0.722658
93	H	0	0.952745	-3.033314	-2.423393
94	C	0	-1.658631	-4.242688	-0.611001

95	C	0	-1.363302	-4.2521	0.901461
96	H	0	-0.308742	-4.051353	1.1131
97	H	0	-1.961957	-3.506391	1.435461
98	H	0	-1.603502	-5.235853	1.328779
99	C	0	-0.79254	-5.329345	-1.289527
100	H	0	-1.039207	-6.317926	-0.878487
101	H	0	-0.962019	-5.379518	-2.371234
102	H	0	0.277411	-5.166123	-1.124846
103	C	0	-3.143944	-4.60458	-0.822158
104	H	0	-3.816323	-3.883816	-0.346099
105	H	0	-3.409941	-4.660748	-1.883076
106	H	0	-3.356256	-5.588174	-0.380777
107	S	0	3.790812	1.023678	-0.967463
108	S	0	-3.79085	-1.0237	-0.967374
109	Si	0	6.003406	-0.96692	-0.03706
110	Si	0	1.228135	2.52939	-1.417205
111	Si	0	-1.228105	-2.529245	-1.417284
112	Si	0	-6.003452	0.966909	-0.037005
